# Tutorial for variant interrogation in tumor samples

**DOI:** 10.1371/journal.pcbi.1013924

**Published:** 2026-02-17

**Authors:** Riley J. Arseneau, Leah K. MacLean, Jeanette E. Boudreau, Daniel Gaston

**Affiliations:** 1 Department of Pathology, Dalhousie University, Halifax, Nova Scotia, Canada; 2 Beatrice Hunter Cancer Research Institute, Halifax, Nova Scotia, Canada; 3 Department of Microbiology and Immunology, Dalhousie University, Halifax, Nova Scotia, Canada; 4 Pathology & Laboratory Medicine, Nova Scotia Health, Halifax, Nova Scotia, Canada; SIB: Swiss Institute of Bioinformatics, SWITZERLAND

## Abstract

The increasing accessibility of next-generation sequencing has empowered researchers to investigate somatic mutations in cancer. The complexity of variant analysis pipelines, terminology, and tool selection remains a major barrier, especially for those new to the field or working in translational settings. To address this challenge, we present a practical framework that guides researchers through the critical steps of variant interrogation in tumor samples. This guide is broken into four phases: *Planning*—laying the foundation for thoughtful experimental design and a clear understanding of sequencing outputs; *Gathering Resources*—assembling the tools, reference data, and variant annotation sets required for analysis; *Filtering and Validation*—executing a systematic approach to prioritize meaningful variants; and *Dissemination and Storage*—ensuring findings are reproducible and accessible through transparent reporting and data sharing. Developed with an emphasis on accessibility, reproducibility, and clinical relevance, this framework equips researchers with the guidance to navigate variant analysis with confidence and rigor.

## Introduction

**Fig 1:**
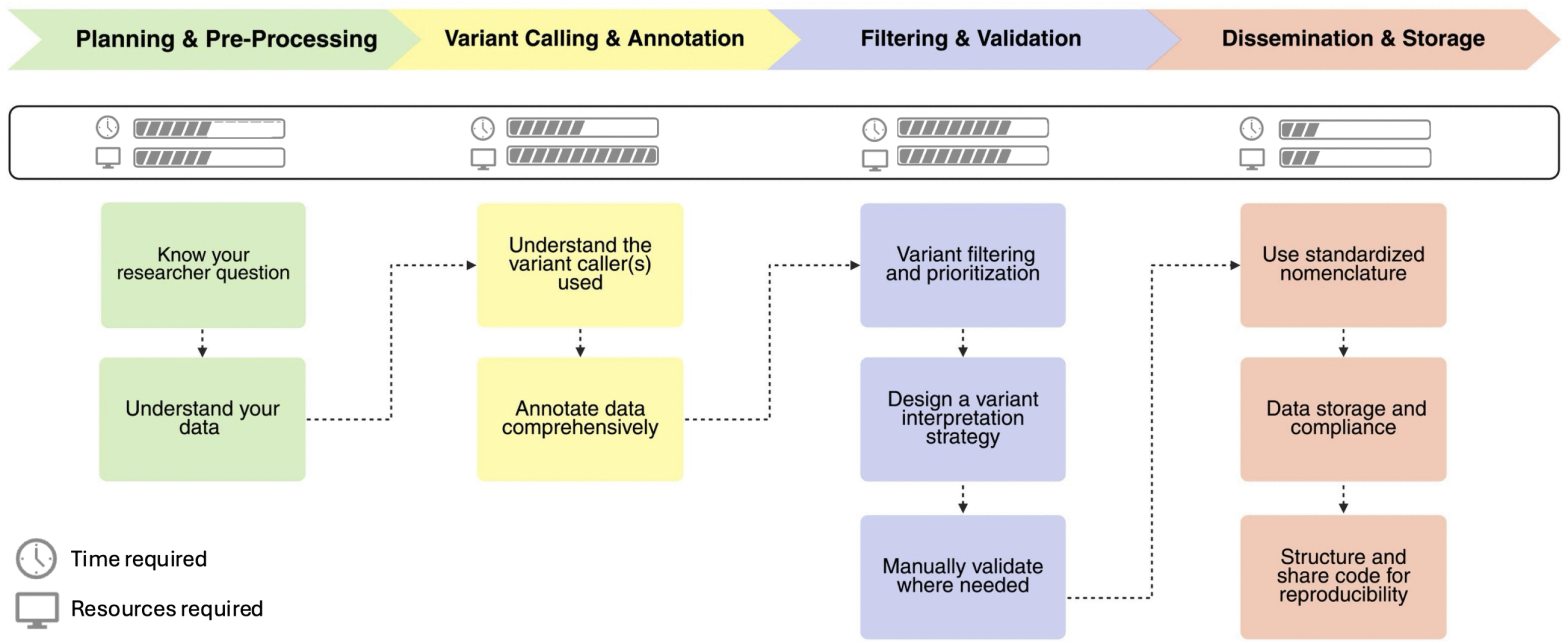
General workflow of variant interrogation in tumor samples. Please note that the steps are intended to be taken sequentially, and each should be completed before moving on to the next; however, depending on how data has been processed prior to your work, it may be necessary to start later in the pipeline. Created in BioRender. Arseneau, R. (2026) https://BioRender.com/armq9mn.

Next-generation sequencing (NGS) enables the investigation of somatic mutations in cancer. However, the concurrent proliferation of analysis pipelines, plugins, and programs [1] can be overwhelming for beginners. This *tutorial for variant interrogation in tumor samples* (Fig 1 provides a practical roadmap for analyzing sequencing data and disseminating findings. It is intended for researchers new to NGS or seeking greater confidence in variant analysis workflows.

We aim to empower researchers to navigate variant interrogation in tumor samples using the tools available publicly. Inspired by clinical guidelines but adapted for translational research, this guide excludes clinical decision making, which requires clinical training, licensure, and stringent criteria [2,3]. Our glossary of key terms and concepts should be reviewed before reading the tutorial (Table 1). Most sequencing analysis, including many of the tools discussed in this article, requires familiarity with the command line interface (CLI); resources are available elsewhere [4]. CLI code examples are provided throughout this manuscript, and working through our demonstration dataset will reinforce key principles (S1 Fig, S1 Data).

**Table 1:** Key terms and concepts.

Concept	Acronym	Definition / Description
***Sequencing approaches***		
Targeted sequencing	TS	Sequencing approach focused on targeted regions of the genome. Targeted sequencing covers highly curated combinations of genomic regions that can include coding, non-coding regions, and/or genes associated with a particular pathology.
Whole genome sequencing	WGS	Yields sequencing data across the entire genome, including both coding and non-coding regions. WGS can identify indels, CNVs, and SNVs. WGS can identify variants that impact splicing patterns or non-coding RNAs.
Whole exome sequencing	WES	Generates sequencing data for all areas of the genome that encode proteins (the exome).
***Variant types***		
Copy number variant	CNV	A type of SV in which a duplication or deletion changes the total number of copies of a DNA region within the genome.
Germline variant		Variants present in germ cells that are passed to all cells within the organism and passed from parent to offspring.
Insertion-deletion variant	Indel	Small insertion or deletion (<50 base pairs) variants.
Single-nucleotide variant	SNV	A variant in which a single nucleotide is substituted for another. SNVs within a coding region can be synonymous (no change in amino acid) or nonsynonymous (causes an amino acid substitution). SNVs can be rare or common within populations of humans of different ancestry.
Somatic variant		Acquired variants are only present in a subset of cells within an organism.
Structural variant	SV	A large (>50 base pairs) rearrangement of part of the genome.
***File formats***		
Annotated variant call format		A variant call format file with additional information about each variant, typically after processing by tools that provide functional annotations, clinical significance, or other details.
Binary alignment map	BAM	The compressed binary format of the SAM file format. Faster to read and write than SAM files. BAM is lossless compression, therefore can convert back from BAM to SAM.
FASTQ		A text-based file format that is an extension of the FASTA file format and stores the sequence ID, the sequence itself, and sequence quality data. Most common format for storing raw sequencing data.
Sequencing alignment map	SAM	Text-based file format for storing sequencing data aligned to a reference genome. SAMs are typically large files that are converted to a BAM file (lossless compressed file).
Variant call format	VCF	Text-based file format that stores SNV, indel, and structural variation calls. VCFs have two main components: the header, which stores information about the dataset and necessary reference files, and the variant call records, which store information about each variant called.
***Sequencing concepts***		
Base quality score		A score used to indicate the probability of an error in a specific base call. This score is represented on a Phred scale.
Phred scaling/scores		A logarithmic score expressing the confidence of a base call or variant call in sequencing. A higher Q indicates a higher confidence. Q10: 1/10 error probability (90% accuracy). Q20: 1/100 error probability (99% accuracy). Q30: 1/1000 error probability (99.9% accuracy). Q40: 1/10000 error probability (99.99% accuracy).
Sequence breadth of coverage (genome coverage)		The proportion of the genome (or targeted region) that has been covered by at least one sequencing read. Ensures the entirety of the targeted region has been sequenced.
Sequence depth of coverage (read depth)		The number of times a specific nucleotide in the genome is read during sequencing OR the average number of times the genome is read during sequencing. Also referred to as read depth. Higher depth of coverage increases the confidence of a called variant at a specific location.
Variant allele frequency	VAF	The frequency at which the variant is detected within the specimen estimated by dividing the number of variant sequencing reads at a particular location by the total number of sequencing reads at that location (reference and variant reads).
Variant functional prioritization		Variant interrogation is used to identify variants with potential or clinical significance. Functional prioritization is also used to identify variants that require manual validation.
Variant quality filtering		Variant filtering is used to distinguish true positive variants from false positive variants.
Variant quality score		A score that reflects the confidence that a detect variant truly differs from the reference genome. This score is represented on a Phred scale.
***Variant analysis concepts and tools***		
Catalogue of Somatic Mutations in Cancer	COSMIC	Comprehensive resource cataloguing the occurrence of somatic mutations in human cancers.
ClinVar		Public archive reporting the relationships among human genomic variations and phenotypes hosted by the National Center for Biotechnology Information (NCBI).
Flag		A label in the filter field of a VCF that indicates if a variant has passed or failed quality control filters.
Genome aggregation database	gnomAD	Database of exome and genome sequencing data to catalog the frequency of variants within human populations.
Indexing		The process of creating an auxiliary file that records the positions of data within a large genomic file, allowing rapid access to specific genomic regions without scanning the entire file.
Integrative genomic viewer	IGV	A visualization tool for interactive exploration of variants and genomic alignments.
LiftOver		The process of converting genomic coordinates from one reference genome to another.
Pathogenicity predictors		A subset of variant annotators that interrogate identified variants to predict if a given variant is deleterious or associated with disease.
Population allele frequency		The proportion of chromosomes in a population that carry a specific allele. This represents how common a particular variant is within a population.
Variant annotators		Any tool that provides information at the variant-level.
Variant effect predictor	VEP	A toolset curated and maintained by Ensembl is used for the analysis, annotation, and prioritization of genomic variants.
Variant callers		Algorithmic tools are used to detect differences between the aligned reads of a sample and the corresponding reference genome.
***Organizations with variant analysis and/or reporting guidelines***		
American College of Medical Genetics Criteria	ACMG	Categorize variants in Mendelian disorders into five categories based on multiple lines of evidence; Pathogenic—Strong evidence that the variant causes disease Likely pathogenic—High likelihood of being disease-causing but not definitive Uncertain significance—Insufficient or conflicting evidence Likely Benign—Likely harmless but not fully confirmed Benign—Strong evidence the variant does not cause disease
Association of Molecular Pathology and American Society of Clinical Oncology Criteria	AMP ASCO	Classify somatic mutations into four tiers based on clinical relevance; Tier 1: Strong clinical significance—FDA-approved therapies Tier 2: Potential clinical significance—Clinical trials, emerging data Tier 3: Unknown Clinical significance Tier 4: Likely benign or neutral
Hugo Gene Nomenclature Committee	HGNC	A working group under the Human Genome Organization, with the aim to define the standard for the description of all DNA, RNA, and protein variants.
Human Genome Variation Society	HGVS	International guidelines for the standard description of DNA, RNA, and protein-level sequencing variants. These guidelines are managed by the HGNC.
***Additional definitions***		
Lossless compression		A form of data compression that reduces file sizes without sacrificing any information in the process.
Lossy compression		A type of data compression used when a file can afford to lose some data. Information is lost during lossy compression that cannot be retrieved when decompressing.
Formalin-fixed paraffin-embedded	FFPE	A common way of preserving tissue samples from which DNA or RNA is often extracted for sequencing.

## Phase 1: Planning and pre-processing

### Tailor the sequencing approach or selection of existing datasets to the research question

Whether generating new data or analyzing existing datasets, the research question(s) determines the most appropriate sequencing type, as methods differ in variant detection [1,5]. An ill-suited method risks poor data [5], while the right approach maximizes relevant variant detection [5]. Key guiding questions include:

- Are you characterizing alterations across the genome, or focusing on specific genes or mutation types (e.g., **single nucleotide variants** (SNVs), **insertions/deletions** (indels), **structural variants** (SVs), or **copy number variations** (CNVs))?
- Are you seeking novel or low-frequency variants?
- Are you interested in coding regions, non-coding regions, or both?

These questions clarify whether **whole genome sequencing** (WGS), **whole exome sequencing** (WES), or **targeted sequencing** (TS) best suits the study. Each involves trade-offs in breadth and depth of coverage, variant detection capability, and cost (Table 2). Higher **depth of coverage**, or read depth, increases confidence in detecting low-frequency variants [6], while **breadth of coverage** reflects how much of the genome is sequenced [7]. Depth is particularly relevant in highly heterogenous samples like tumors, where high variability and low-frequency mutations are expected [6]. Higher depth increases cost and computational requirements; WES/TS offer higher depth over smaller regions, while WGS provides broad coverage at lower per-region depth [1]. While each approach can detect the types of variants listed in Table 2, their sensitivity varies (e.g., CNV and SV detection with WES and TS is limited by capture biases and uneven coverage) [1,8,9].

**Table 2:** Summary of sequencing approaches for variant detection.

Sequencing Approach	Average cost per sample*	Breadth of Coverage	Minimum Read Depth (Min-Recommended)	Detectable Variants
Whole genome sequencing	$$$	~95–98% of the genome	30–40× (germline) 80–100× (tumor)	SNVs, Indels, CNVs, SVs
Whole exome sequencing	$$	~1–2% of genome; ~85–95% of exome	100–200× (tumor)	SNVs, Indels CNVs
Targeted sequencing	$	<<1% of genome; ~100% of targeted regions	500–1000× (tumor)	SNVs, Indels, CNVs

Comparison of sequencing approaches used in tumor genomic profiling. Each approach offers trade-offs in cost, resolution, and variant detection capability. Values are derived from Illumina [12] and Yu and colleagues, 2023 [13] and will vary with adaptations to best practices as cost of sequencing decreases.

Long read sequencing technologies are increasingly incorporated into cancer genomic studies [10]. While they offer improved SV detection and resolution of complex regions, they have higher error rates, lower throughput, and greater cost compared to short-read platforms [10,11].

Even with a clear strategy, practical constraints may necessitate compromise. Considerations include:

- Tumor content: Samples with <30% tumor cells require greater sequencing depth due to reduced sensitivity [14].
- Sample quality: Fresh frozen samples generally yield high-quality DNA, while **formalin-fixed paraffin-embedded** (FFPE) tissues often require higher depth to account for artifacts [15].
- Budget: WGS costs most per sample, despite having the lowest cost per base pair.
- Computational resources: requisite processing power is directly proportional to the amount of data; narrowing breadth of coverage reduces data burden.
- Control samples: Control samples (e.g., commercial samples [16], panel of normals, germline samples) [17–19] improve confidence of variant calling [20].
- Public dataset (e.g., The Cancer Genome Atlas [21]) availability may necessitate adaption of the research objectives.

### Understand the capabilities of the sequencing data used

Understand the sequencing data type and its processing history. Data may be received at any stage in the processing pipeline, with each step involving different files, tools, and assumptions. Incomplete or overprocessed data can lead to false positives, missed variants, and irreproducible results [22,23].

**Fig 2:**
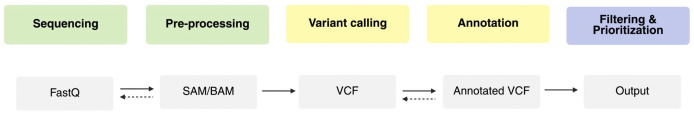
Flowchart of common sequencing file types and analysis stages. The diagram illustrates the typical file types encountered throughout the sequencing and analysis pipeline, progressing from raw data (left) to results (right). File types are grouped by processing phase: green (Phase 1: Planning and pre-processing), yellow (Phase 2: Variant calling and annotation), and blue (Phase 3: Filtering and validation). Solid arrows indicate the standard forward progression of file generation, while dotted arrows represent steps where data can be reverted to a previous file type. The objective is to complete the pipeline, transforming raw reads into interpretable variants. Created in BioRender. Arseneau, R. (2026) https://BioRender.com/wx6ql68.

The typical workflow includes pre-processing/alignment, variant calling, annotation, filtering, and prioritization (Fig 2). Table 3 outlines common genomic file type structures (e.g., **FASTQ**, **sequencing alignment map** (SAM)/ **binary alignment map** (BAM), **variant call format** (VCF), and **annotated VCFs**).

**Table 3:** Common sequencing file types and their structure.

File Type	Structure
FASTQ [24]	Each read contains: 1. Sequence identifier 2. Raw sequence nucleotides 3. Optional comment line(s) 4. Quality score strings
SAM [25]	Contains a header followed by alignment reads: 1. QNAME (query name or read identifier) 2. FLAG (bitwise flag for alignment information) 3. RNAME (reference sequence name) 4. POS (1-based leftmost position of the alignment) 5. Other options fields
BAM [25]	Same structure as SAM but compressed in binary format. BAM files are indexed to allow quick retrieval of alignments overlapping specific genomic regions.
VCF/Annotate VCF [26]	Contains a header (metadata and description of each column) followed by data rows for each variant called: 1. CHROM (chromosome) 2. POS (position) 3. ID (variant identifier) 4. REF (reference allele) 5. ALT (alternate allele) 6. QUAL (quality score) 7. Other optional fields If annotated, additional INFO fields are added for annotation data.

While the typical structure of each file type is described, there may be variation in the presence or extent of included metadata.

**Questions to understand previous processing:**

- FASTQ: Are the reads raw or processed (e.g., adaptor trimmed)? Which tools and/or thresholds were used?
- SAM/BAM: Has alignment been performed? Were they aligned to a modern reference genome?
- VCF: Which variant caller(s) was used? Were any filtering parameters applied?
- Annotated VCF: What annotations were applied? Are they suitable for your goals? Were any filters applied that could limit variant output?

**CLI Example Code 1. FASTQ pre-processing.**

# Quality check raw FASTQ files using FastQC

# INPUT: sample_R1.fastq.gz and sample_R2.fastq.gz (paired-end reads)

# OUTPUT: HTML and.zip reports in qc_reports/ directory

fastqc sample_R1.fastq.gz sample_R2.fastq.gz -o qc_reports/

# Align reads to GRCh38 reference genome using BWA-MEM

# INPUT: FASTQ files (R1 and R2), reference genome GRCh38.fa

# OUTPUT: unsorted SAM stream

bwa mem GRCh38.fa sample_R1.fastq.gz sample_R2.fastq.gz -o sample.sam

# Convert SAM to BAM using samtools view

# INPUT: sample.sam

# OUTPUT: sample.bam

samtools view -@ 8 -bS -o sample.bam sample.sam

# Sort BAM file by genomic coordinates

# INPUT: sample.bam

# OUTPUT: sample_sorted.bam

samtools sort -@ 8 -o sample_sorted.bam sample.bam

# Index BAM for fast retrieval in downstream tools

# INPUT: sample_sorted.bam

# OUTPUT: sample_sorted.bam.bai (index file)

samtools index sample_sorted.bam

## Phase 2: Variant calling and annotation

### Understand the variant caller(s) used

Variant calling transforms sequencing data into a list of genetic changes and is typically the most computationally demanding step [27]. While DRAGEN [28], MuTect2 [29], and GATK HaplotypeCaller [30] are widely used callers for detecting SNVs and Indels [22,31], they are not optimal for all sample types or goals. Challenging samples or specialized analyses may require adjusting thresholds or selecting alternate callers [32]. Adjustable caller settings, such as minimum read depth, **base quality score** thresholds, **variant allele frequency (VAF)** cutoffs, and **variant quality scores**, should match the study’s goals; inappropriate thresholds risk false negatives or false positives [33]. Table 4 outlines several variant callers and their typical use cases.

**Table 4:** Variant Caller information.

Variant Type	Specializations Suggested for Variant Caller	Variant Callers
SNV and small indels (<50 bp)	• High-sensitivity tools required for low-VAF detection, especially in somatic contexts [28,29,34]. • Precise base-level resolution and variant quality annotation [28,29,34,35].	Mutect2 [29], VarScan2 [34], FreeBayes [35], DRAGEN [28]
CNVs	• Use of read-depth modeling and/or segmentation algorithms [28,36–38]. • Sensitivity to large-scale copy number changes [28,36–39]. • Ability to integrate matched normal or population controls to reduce false positives [28].	DRAGEN-CNV [28], CNVKit [36], Delly [40], Lumpy [39], CNVnator [37], Canvas [38]
SVs	• Use of split-read and discordant paired-end read signals for breakpoint detection [28,40–43]. • Ability to detect large insertions, deletions, translocations, inversions, and complex rearrangements [28,36,40–42]. • Integration of read depth and mapping anomalies [37,40,41,43].	Pindel [41], DRAGEN-SV [28], Manta [42], Delly [40], CNVnator [37], TIDDIT [43]

Summary of variant types, key considerations, and example callers.

**CLI Example Code 2. SNV calling with MuTect2**

# Call somatic variants using GATK Mutect2

# INPUT: tumor BAM, reference genome GRCh38.fa

# OUTPUT: somatic.vcf.gz (compressed VCF of called variants)

gatk Mutect2 \

-R GRCh38.fa \           # reference genome

-I tumor.bam \             # tumor sample BAM

-O somatic.vcf.gz       # output VCF file

Key considerations when selecting and configuring variant caller(s):

- Variant type: Tools vary in sensitivity to detect different types of variants. Comparative studies [44,45] and documentation can guide selection. Note that SNV and CNV calling can be inconsistent between tools, so validation and cross-caller consensus may be necessary [22]. Consensus calling reduces false positives but may exclude true variants, so it is generally best used for validation or high-confidence reporting, rather than exploratory analyses.
- Sample heterogeneity: Highly heterogenous tumors may require lowering VAF or read depth thresholds to capture subclonal variants [46].
- Sample quality: FFPE DNA is prone to artifacts (e.g., cytosine deamination (C > T) transitions) [15]. Minimize false positives by increasing quality thresholds [15,46].
- Discovery vs. validation: For exploratory analysis, relaxing filtering parameters and/or using multiple variant callers can maximize sensitivity. For validation, stricter filters may be warranted.

Variant callers require alignment to an up-to-date reference genome (e.g., National Library of Medicine [47] or Ensembl [48,49]. If SAM/BAM or FASTQ files are available, it’s best practice to re-align to a modern reference genome. If only VCF files are available, coordinates can be converted between assemblies with **LiftOver** tools (e.g., BCFtools/liftover, CrossMap [50,51]) for better annotation.

**CLI Example Code 3. Cross Caller Consensus Using BCFtools**

# Intersect variants from two callers using bcftools isec

# INPUT: VCF from caller 1 (c1.vcf) and caller 2 (c2.vcf)

# Use bcftools isec to find variants detected by BOTH tools.

# OUTPUT: consensus_output/ directory containing:

#   0000.vcf - > intersection of both callers

#   0001.vcf - > unique to first file (c1.vcf)

#   0002.vcf - > unique to second file (c2.vcf)

#   sites.txt - > list of positions considered in the comparison

# NOTE: -n = 2 ensures only variants present in both files are included in 0000.vcf.

bcftools isec -n=2 c1.vcf c2.vcf -p consensus_output/

**CLI Example Code 4. LiftOver with CrossMap**

# Convert VCF coordinates from GRCh37 to GRCh38 using CrossMap

# INPUT: chain file (GRCh37_to_GRCh38.chain), VCF file, reference genome

# OUTPUT: somatic_lifted.vcf

CrossMap.py vcf GRCh37_to_GRCh38.chain somatic_filtered.vcf.gz \

GRCh38.fa somatic_lifted.vcf

### Annotate data comprehensively

Annotation adds biological context, enables prioritization of meaningful variants, and reduces the need for manual review. Variant annotations can be associated with a specific variant (e.g., *KRAS* c.34G > T, p.G12C), groups of related variants at the same codon (e.g., *KRAS* codon 12 mutations: G12C, G12D, G12V), gene (e.g., all pathogenic variants in the *KRAS* gene*)*, or broader regions of the genome (e.g., SVs or amplifications spanning the *KRAS* locus on 12p12.1) [52].

Annotations are obtained through **variant annotators**, commonly via the CLI or alternatively, web-based platforms. Ensembl’s **Variant Effect Predictor** (VEP) [53] is widely used, offering annotations like population frequencies, clinical significance, and predicted pathogenicity. ANNOVAR [54] and SnpEff [55] are popular alternatives.

Several annotation sources are particularly relevant for somatic cancer analysis. Population allele frequency databases (e.g., **gnomAD** [56] and TOPmed [57]) help exclude common germline polymorphisms. **Pathogenicity predictors** estimate the impact of variants on protein function or gene regulation (Table 5). Curated databases, including **ClinVar** [58], VarSome [59], Franklin by GenoOx [60], OncoKB [61,62], Genomenon Cancer Knowledgebase (Formerly JaxKB) [63], and the **Catalogue of Somatic Mutations in Cancer** (COSMIC) [64] consolidate expert-reviewed literature, functional data, and clinical annotations. COSMIC data are freely accessible for academic use following registration. In the demo dataset provided with this tutorial, the Genome Screens Mutant dataset was used. Beyond these resources, specialized annotations from the literature or pathway databases can offer insight into drug response, regulatory impact, or broader pathways.

**Table 5:** Commonly used pathogenicity predictors.

Tool Name	Variant Type	Tool Purpose	Common Threshold for Pathogenicity
Combined Annotation Dependent Depletion (CADD) [66]	SNV Indel Coding Non-coding	• Scores deleteriousness of variants. • Integrates multiple annotations into one metric. • C-scores increase with increasing variant deleteriousness.	C-score = >15 or >20
ClinPred [67]	Missense SNV	• Focus on disease-relevant non-synonymous variants. • Integrates multiple annotations, including ClinVar. • ClinPred score increases with increasing variant pathogenicity.	ClinPred score ≥0.5 or ≥0.7
BayesDel [68]	SNV Indel Coding Non-coding	• Integrates multiple annotations into one metric. • BayesDel score increases with increasing predicted pathogenicity. • Scores range from −1.29334 to 0.75731.	BayesDel score > -0.0570105
Rare Exome Variant Ensemble Learner (REVEL) [69]	Missense SNV	• Integrates multiple annotations into one metric. • REVEL score ranges from 0 to 1 where higher scores indicate greater likelihood that the variant is deleterious.	REVEL score = ≥0.5 or ≥0.75
MetaLR [70]	Missense SNV	• Integrates nine independent variant deleteriousness scores with variant allele frequency information to predict missense variant pathogenicity. • MetaLR scores range from 0 to 1 where variants with higher scores are more likely to be deleterious. • Variants are categorized as damaging or tolerated.	D = damaging T = tolerated / = N/A
AlphaMissense [71]	Missense SNV	• Combines structural context and evolutionary conservation to predict variant pathogenicity. • AlphaMissense score used to categorize variants into three groups: likely benign, ambiguous, likely pathogenic.	3 variant groups: likely benign (<0.34) ambiguous (0.34-0.564) likely pathogenic (>0.564)
SpliceAI [72]	Splice site variants	• Robust tool for predicting splicing defects caused by DNA variations. • Delta score increases with the likelihood of splicing defects.	Delta score = >0.5 or >0.8

Summary of computational tools used for pathogenicity prediction. Description of tools used for pathogenicity predictions, the variant types for which they are appropriate, and the thresholds recommended by each tool to indicate pathogenicity.

Comprehensive annotation is important; however, excessive annotations can inflate file sizes and complicate variant filtering or interpretation. Select complementary resources that align with your research objectives [52] using recent literature and tool or database documentation [52,65].

**Example CLI Code 5. Annotation with VEP**

# Annotate variants using Ensembl VEP

# INPUT: somatic.vcf.gz

# OUTPUT: somatic_annotated.vcf with annotations

vep \

--input_file somatic.vcf.gz \

--output_file somatic_annotated.vcf \

--cache \

--assembly GRCh38 \

--vcf

## Phase 3: Variant filtering and validation

### Filter and prioritize candidate variants

After variant annotation, reduce the variant list by **quality filtering** (remove unreliable variants [73,74]), and **functional prioritization** (elevate those most likely to be biologically or clinically relevant [75]).

#### Quality filtering.

Quality filtering uses caller metrics and sequencing parameters to remove artifacts. Here we discuss filtering considerations; however, thresholds will vary by dataset. During variant calling, variants receive a “PASS” FILTER **flag** if they meet all the caller’s quality requirements. Alternative FILTER field flags are defined by individual variant callers, described in the output VCF header or in the software documentation. Retaining only PASS flags may exclude true variants, but including non-PASS variants risks admitting artifacts. Publicly available VCFs are often pre-filtered.

**VAF** often informs PASS criteria. Depending on the assay’s detection limit, additional VAF-specific filtering may be necessary. Typical somatic cancer minimum VAF thresholds are 5%–10% of total reads [76–78]; however, dynamic thresholds can be used to account for variability in depth of coverage. Variants observed at highly similar VAFs across many samples may indicate run-specific artifacts [79]. Control samples with known VAFs can help empirically define the lower limit of detection for the sequencing run.

Variant callers aggregate base quality scores (Phred-scaled, 30 = 99.9% confidence [80]) and other signals to estimate confidence in the variant as a **variant quality score**. Minimum scores of 30 are commonly used to balance sensitivity and specificity [81,82].

#### Functional prioritization.

Functional prioritization ranks variants by biological or clinical relevance using annotations, either within annotation tools (e.g., Ensembl’s VEP) [53] or *post hoc*. Functional prioritization follows either clinical-grade binning or research-focused prioritization [2,3,75].

Clinical frameworks from organizations like the **American Society of Clinical Oncology** (ASCO) [2] and the **American College of Medical Genetics and Genomics** (ACMG) [3] classify variants into tiers or pathogenicity categories. These strategies are aimed at clinical decision-making, as their high stringency may omit variants that could be of interest in research.

**Fig 3:**
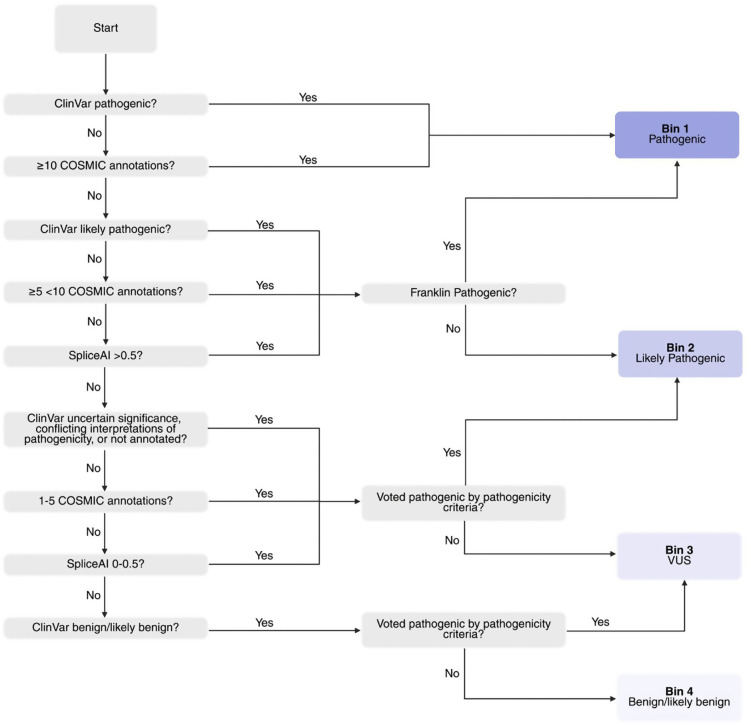
Example variant prioritization scheme. The flowchart illustrates a strategy for prioritizing variants into four bins based on predicted pathogenicity. Variants are initially assigned to a bin using criteria including ClinVar annotations, COSMIC frequency, and SpliceAI scores. Variants may then be reclassified to a different bin based on additional pathogenicity criteria, such as Franklin classifications (for promotion from Bin 2 to Bin 1), or other computational predictors including CADD, REVEL, and phastCons conservation scores (for promotion from Bin 4 to Bin 3, or Bin 3 to Bin 2). Bin 1 contains pathogenic variants, Bin 2 contains likely pathogenic variants, Bin 3 contains variants of uncertain significance (VUS), and Bin 4 represents likely benign or benign variants. Created in BioRender. Arseneau, R. (2026) https://BioRender.com/o4pbmzu.

Research prioritization strategies weigh features like predicted functional impact, evolutionary conservation, presence in known cancer gene lists or curated databases, and occurrence within your cohort or in public datasets [75]. Fig 3 illustrates an example prioritization scheme.

During prioritization, common germline polymorphisms are excluded using population databases (e.g., gnomAD [56] and TopMED [57] with typical cutoffs of 0.01%–1% [83], while curated tumor lists can be used to help identify expected versus novel variants [75]. Most prioritization strategies emphasize protein-coding variants; however, adjust prioritization of non-coding or regulatory variants if of interest.

### Employ a robust variant interpretation strategy

Interpretation integrates annotations, literature, databases, and prior knowledge to generate biologically meaningful hypotheses. Passing filters does not make a variant meaningful; a variant must relate to pathology by impacting gene expression, protein structure or function, regulatory mechanisms, or downstream molecular pathways [75].

Cancer interpretation often focuses on oncogenes with activating mutations or amplifications [84], or tumor suppressor genes, which exhibit deletions, truncations, or inactivating mutations [85]. ClinVar [58] and COSMIC [64] remain central repositories for variant-level information. Online databases (e.g., OncoKB [62], VarSome [59], Franklin [60], and the Clinical Interpretation of Variants in Cancer [86]) provide additional information, including clinical significance, relevant publications, ACMG/ASCO classifications, pharmacogenomic associations, and community-submitted interpretation. Pathway analysis and Gene Set Enrichment Analysis [87,88] can reveal broader relevance for variants that may appear marginal in isolation. The list of molecular changes relevant to cancer continues to expand; thus, a comprehensive literature review is essential [89]. Ultimately, your scientific judgement is essential for variant interpretation.

### Manually validate variants where appropriate

Pipeline quality controls may miss false positives, so manual review is essential for confirming variants. Tools like **Integrative Genomics Viewer** (IGV) [90] allow inspection of read alignment, the variants position within reads, and local CNV [91]. IGV is essential for novel or unexpected findings, and guidelines are available elsewhere [91].

For paired normal-tumor sequencing, both sequencing alignments (tumor and normal) should be evaluated to confirm somatic status [22,91]. Similarly, when a control sample has been sequenced, it should be compared with the sample of interest.

## Phase 4: Disseminating and storage

### Use standardized nomenclature

When disseminating results, standardized nomenclature ensures variants are universally understandable, traceable to reference data, and correctly interpreted [2,92] (Table 6).

**Table 6:** Variant reporting checklist.

Checklist items	Complete?
HGNC-approved DNA, RNA, and protein symbols are used consistently and correctly formatted.	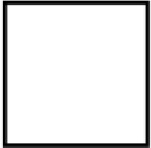
Transcript(s) used for variant annotation are clearly specified (preferably MANE). If MANE transcripts are unavailable, document the transcript and rationale.	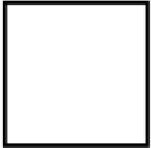
Variant descriptions have been externally validated.	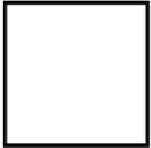
A table with DNA (including genomic coordinates), RNA, and protein information for reported variants is included in the text or supplementary data.	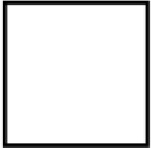

#### Gene and protein nomenclature.

Use gene and protein symbols from the **Human Genome Organization (HUGO) Gene Nomenclature Committee** (HGNC) [92], maintaining one consistent name and introducing aliases at first mention (e.g., *CD274*, a.k.a. *PDL1* or *B7H1*) [92]. Specify the reference transcript used for annotation, preferably the Matched Annotation from NCBI and EMBL-EBI (MANE) [93].

#### Variant reporting.

Report variants at the DNA level following **Human Genome Variation Society** (HGVS) guidelines [94]. Present designations in both the manuscript text and a table that includes DNA, RNA, and protein nomenclature where applicable [95]. Verify variant descriptions with tools like Mutalyzer [96] for HGVS compliance and formatting [97].

**CLI Example Code 6. Verifying variant descriptions with Mutalyzer**

# Normalize an HGVS description (use canonical form for reporting)

# INPUT: Genomic or transcript HGVS (e.g., “GRCh38 (chr<CHR>):g.<POS><REF>><ALT>” or “NM_<TRANSCRIPT>:c.<...>”)

# OUTPUT: JSON with normalized_description (report this)

curl -sS “https://v3.mutalyzer.nl/api/normalize/<YOUR_HGVS_DESCRIPTION>”

# Map a normalized description to a specific transcript (e.g., MANE Select)

# INPUT: description=<YOUR_NORMALIZED_HGVS > ; target_selector=<TRANSCRIPT_ACCESSION> (e.g., NM_########.#)

# OUTPUT: JSON with c.-notation for the requested selector

curl -sS --data-urlencode “description=<YOUR_NORMALIZED_HGVS>” \

--data-urlencode “target_selector=<TRANSCRIPT_ACCESSION>” \

“https://v3.mutalyzer.nl/api/map/”

# List available selectors (transcripts) for a reference sequence

# INPUT: reference_id=<REFERENCE_ACCESSION> (e.g., NC_########.##)

# OUTPUT: JSON array of selector IDs (choose MANE when available)

curl -sS https://v3.mutalyzer.nl/api/get_selectors/<REFERENCE_ACCESSION>

# Convert reference positions to selector-oriented coordinates (genome to c.-notation)

# INPUT: Genomic HGVS (e.g., “GRCh38 (chr<CHR>):g.<POS><REF>><ALT>” or “NC_########.##:g.<...>”)

# OUTPUT: JSON with c.-level coordinates aligned to the chosen selector

curl -sS --data-urlencode “description=<YOUR_GENOMIC_HGVS>” \

“https://v3.mutalyzer.nl/api/position_convert/”

### Genomic data storage and compliance

Genomic data management should follow Findable, Accessible, Interoperable, and Reusable principles (FAIR) [98].

#### Working directory storage.

During active analysis, use hierarchical directory system for raw data, intermediate files, results, and metadata [99]. Apply a version control system to track changes in scripts and metadata [98].

#### Long-term storage.

Retain files essential for future auditing, reanalysis, or validation [100], including raw data, analysis scripts, auxiliary files, and selected results. Genomic files are large, making compression essential for storage. Two primary types of compression are available: lossless and lossy. **Lossless** formats like FASTQ.gz and BAM are preferred for permanent storage. When **lossy** compression is used, its impact on downstream analyses should be considered [101]. All transformations should be logged with details on software, versions, and parameters [98].

#### Storage scalability, redundancy, and security.

Combine institutional servers, cloud storage, and external drives for redundancy [99,100,102] and ensure compliance with ethical, legal, and institutional standards [103], including encryption and secure transfer protocols [100,104]. Deposit data in secure external repositories when possible [98].

### Structure and share code for reproducibility

Genomic analyses rely on complex workflows that must be documented for reproducibility, validation, and reuse [105] (Table 7). Share code when possible [98,105] via repositories such as GitHub [106] or GitLab [107]. Use workflow managers (e.g., Snakemake [108], Nextflow [109]) and package/container managers (e.g., Conda [110], Docker [111]) to standardize environments and automate pipelines [112]. Include README files detailing file structures, workflows, and expected outputs [100,113]. Code availability statements can be referenced from *The American Journal of Human Genetics* [114] or *Oxford Academic* [115].

**Table 7:** Code reproducibility and sharing checklist.

Checklist items	Complete?
Archive the finalized scripts, workflows, software versions, and the date of dataset download alongside all datasets used in the analysis.	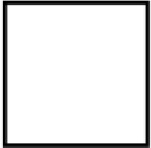
Write well-annotated code that can be easily adapted to the environments of other users. Prioritize modular structures and functions and avoid hard-coded path names.	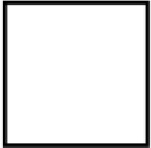
Use version control systems, such as Git [116], along with external repositories to maintain code integrity.	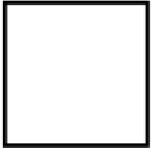
A include README and configuration files with details about the computational environment to ensure replicability.	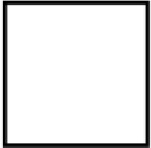

## Conclusions

This framework provides practical guidance for tumor sequencing analysis, covering the full workflow—from study design to data interpretation and dissemination—while emphasizing code sharing to foster reproducibility and collaborative science. By promoting a structured, reproducible approach, these guidelines support consistency in variant interpretation and reporting, contributing to greater clarity, transparency, and comparability across studies in cancer genomics.

## Supporting information

S1 Fig Variant interrogation in practice: step-by-step questions using demo data. Guiding questions for demo data used to demonstrate variant filtering and prioritization in tumor and control samples. Box 1 focuses on interrogating the dataset in question. Box 2 focuses on variant annotations and their use cases. Box 3 and Box 4 encompass quality filtering and functional prioritization of variants. Box 5 guides the identification and reporting of final variants of interest. Box colors correspond to processing phase: green (Planning and pre-processing), yellow (Variant calling and annotation), and blue (Filtering and validation). Created in BioRender. Arseneau, R. (2026) https://BioRender.com/gexi4i4.
(TIF)

S1 Data Example data and associated code for working through variant annotation and interrogation following the guidelines laid out in this manuscript. (ZIP)
